# Meditation for subjective cognitive decline, mild cognitive impairment and Alzheimer’s disease: a systematic review and meta-analysis of randomized controlled trials

**DOI:** 10.3389/fpubh.2025.1524898

**Published:** 2025-05-30

**Authors:** Jiaxin Shi, Hao Tian, Jingwen Wei, Wenhan Xu, Qin Luo, Jin Peng, Jun Xia, Wenying Huai, Ying Xiong, Yunhui Chen

**Affiliations:** ^1^CDUTCM-KEELE Joint Health and Medical Sciences Institute, School of Acupuncture and Tuina, School of Basic Medical Sciences, Chengdu University of Traditional Chinese Medicine, Chengdu, Sichuan, China; ^2^West China Hospital, Sichuan University, Chengdu, Sichuan, China

**Keywords:** meditation, subjective cognitive decline, mild cognitive impairment, Alzheimer’s disease, meta-analysis

## Abstract

**Background:**

Meditation has gained increasing recognition as a simple, cost-effective, and non-invasive therapeutic approach for older adults with subjective cognitive decline (SCD), mild cognitive impairment (MCI), and Alzheimer’s disease (AD). This meta-analysis aimed to systematically evaluate its effectiveness on this population.

**Methods:**

A comprehensive search across nine databases was performed from inception to April 1, 2024, to identify eligible randomized controlled trials (RCTs). The primary outcome was global cognitive performance measured by the Mini-Mental State Examination (MMSE), while the secondary outcomes included sleep quality estimated through the Pittsburgh Sleep Quality Index (PSQI), health status assessed using the 36-Item Short Form Health Survey (SF-36), and depression evaluated with the Geriatric Depression Scale (GDS). This meta-analysis utilized R 4.3.1 software and adhered to the Cochrane Handbook and PRISMA reporting guidelines.

**Results:**

A total of 25 RCTs published between 2013 and 2024 involving 2,095 participants were included in this study. The pooled findings demonstrated that meditation significantly improved global cognitive performance (MD 2.22, 95% CI: 0.83–3.62, *p* = 0.002), sleep quality (MD −1.40, 95% CI: −2.52 to −0.27, *p* = 0.015), and health status (MD 3.50, 95% CI, 0.45–6.56, *p* = 0.020). However, no significant effect was observed on depression compared to the control group (SMD −0.16, 95% CI: −0.63 to 0.31, *p* = 0.514).

**Conclusion:**

This meta-analysis suggests that meditation is an effective adjunct therapy for improving global cognitive performance, sleep quality, and health status in older adults with SCD, MCI, and AD. However, given the heterogeneity and limited sample sizes, these findings should be interpreted with caution. More large-scale and high-quality RCTs are needed to further substantiate these effects.

## Introduction

With the global rise in population aging, mild cognitive impairment (MCI) and Alzheimer’s Disease (AD) have emerged as major contributors to disability worldwide representing a continuum of cognitive disorders associated with aging and neurodegeneration, particularly under the broader spectrum of AD progression (1). Within this continuum, subjective cognitive decline (SCD), characterized by self-reported cognitive complaints in the absence of objective impairment, is increasingly recognized as the earliest clinical manifestation of AD pathology. SCD is associated with amyloid-β (Aβ) accumulation biomarkers and neurodegeneration, conferring an elevated risk of progression to MCI and subsequent dementia. Epidemiologically, cognitive decline has been rising dramatically in recent years and is estimated to reach 115 million cases by 2050, and 30–50% of individuals with SCD may progress to MCI within 4–5 years with a substantial proportion eventually advancing to AD (2, 3). Given the socioeconomic and public health burden imposed by these conditions, the development and implementation of evidence-based therapeutic strategies are urgently warranted (4).

Current therapeutic options for cognitive decline remain limited. Although pharmacotherapies may delay cognitive decline by 6–12 months in about 50% of AD patients (3),^1^ their clinical utility is constrained by transient efficacy and numerous adverse events. Furthermore, no disease-modifying therapies are available, necessitating a paradigm integrating non-pharmacological interventions to alleviate this pressing public health challenge. Among these, meditation, encompassing modalities such as focused attention meditation (FAM) and dynamic mindfulness meditation (DMM), demonstrates significant potential to improve cognitive performance, concentration, and long-term memory in older adults (5–7), while concurrently reducing sleep disturbances, enhancing mental health, and improving overall quality of life (8). Additionally, its cost-effectiveness and safety also facilitate its scalability in clinical and community settings for widespread use, including nursing homes (7, 9, 10).

Emerging evidence suggests that meditation may mitigate neurocognitive decline by regulating neuroinflammation and promoting neuroplasticity. Studies in individuals with MCI and AD have revealed that sustained meditation practice induces structural and functional brain adaptations, including increased cortical thickness and gray matter volumes in regions critical for executive function, memory consolidation, and emotional regulation (11, 12). Physiologically, meditation attenuates the sympathetic nervous system, modulates the hypothalamic–pituitary–adrenal axis, and enhances parasympathetic nervous system activity, contributing to a neuroprotective state of relaxation and enhancement of patient wellbeing (13). Biomarker studies further indicate that meditation may improve plasma Aβ levels and modulate telomere length and attrition, which correlate with enhanced cognitive performance, sleep quality, mood, and overall quality of life (QOL) (14, 15). These findings collectively suggest that meditation may yield a promising non-pharmacological intervention to enhance brain structure and function, regulate neurodegenerative-related biomarkers, and improve cognitive and overall wellbeing in the aging population.

In recent years, randomized controlled trials (RCTs) investigating the therapeutic potential of meditation in individuals with cognitive decline have been steadily increased. Although pervious reviews have provided valuable insights into this topic, their scope has been primarily focused on cognitive outcomes and are constrained by their reliance on older studies (3, 7). To advance an updated and comprehensive synthesis of the evidence, we conducted a systematic review and meta-analysis integrating the “Reach,” “Effectiveness,” and “Implementation” dimensions of the RE-AIM framework into the research design (16, 17). In this study, the impact of meditation was assessed on global cognitive performance, sleep quality, health status, and depression across the spectrum of SCD, MCI, and AD. Hopefully, these findings may yield an empirical foundation for developing public health strategies to integrate meditation into clinical and community-based care paradigms for aging populations.

## Methods

This systematic review was conducted in adherence to the PRISMA reporting guidelines (18). The study protocol was registered with PROSPERO (CRD42019145932) and published^2^ prior to the initiation of the research (19). No ethical approval was required, as it was a secondary analysis of de-identified data.

### Changes to the study protocol

Before conducting the study, the protocol was modified as follows: (1) The study population was expanded to include individuals with SCD in addition to MCI and AD. This adjustment aligned with emerging research since 2020 highlighting SCD as a critical precursor to MCI and AD, thereby enhancing the public health significance of this study. (2) Outcome measures were streamlined to prioritize consistently available and representative assessments, as certain measures in the original protocol (e.g., Alzheimer’s Disease Assessment Scale-Cognitive Subscale, Activities of Daily Living Scale, Trail Making Test, Stroop Test, Digit Span, Hopkins Verbal Learning Test, Rey Auditory Verbal Learning Test, California Verbal Learning Test II, Rey Complex Figure Test, Clock-Drawing Task, Lowenstein Occupational Therapy Cognitive Assessment, Boston Naming Test, improvements in biomarkers, and effective rates) were inconsistently or insufficiently reported across RCTs. This refinement was to strengthen the rigor and feasibility of this study.

### Data source

Comprehensive searches without language restriction were implemented across nine databases, including PubMed, EMBASE, Web of Science (WoS), Cochrane Central Register of Controlled Trials (CENTRAL), World Health Organization International Clinical Trials Registry Platform (WHO ICTRP), China National Knowledge Infrastructure (CNKI), China Biology Medicine (CBM), China Science and Technology Journal Database (VIP), and Chinese Clinical Trial Registry (ChiCTR) from the inception to April 1, 2024. The detailed search strategy is presented in Table 1, with appropriate amendments applied to other databases as needed.

**Table 1:** Search strategy for the PubMed.

#1 Alzheimer’s disease [Mesh]
#2 AD
#3 Alzheimer∗
#4 MCI
#5 Mild cognitive impairment
#6 Cognitive impairment
#7 Cognitive disorders
#8 Subjective Cognitive Decline
#9 Cognitive Declines
#10 Primary senile degenerative
#11 Cognitive dysfunction
#12 Neurocognitive disorder
#13 Mild neurocognitive
#14 Cognitive decline
#15 #1 OR #2 OR #3 OR #4 OR #5 OR #6 OR #7 OR
#8 OR #9 OR #10 OR #11 OR #12 OR #13 OR #14
#16 Meditation [Mesh]
#17 Metta meditation
#18 Mindfulness
#19 Mindfulness Training
#20 Mindfulness-Based Intervention
#21 Kundalini
#22 Zen meditation
#23 Zazen meditation
#24 Transcendental meditation
#25 Kirtan Kirya
#26 Mantra
#27 #16 OR #17 OR #18 OR #19 OR #20 OR #21
OR #22 OR #23 OR #24 OR #25 OR #26
#28 randomized controlled trial [pt]
#29 controlled clinical trial [pt]
#30 randomized [tiab]
#31 randomly [tiab]
#32 trial [tiab]
#33 groups [tiab]
#34 #28 OR #29 OR #30 OR #31 OR #32 OR #33
#35 #15 AND #27 AND #34

### Eligibility criteria

Only RCTs were included in this review, while observational and longitudinal studies were excluded. The study population comprised SCD, MCI, and AD patients diagnosed with internationally recognized guidelines (20). The meditation interventions encompassed various meditation practices, such as mindfulness-based stress reduction (MBSR), Metta, Mantra, Zen, Kirtan Kriya, Kundalini, and Tibetan Sound Meditation. Control groups included active and non-active comparators, such as usual care, cognitive rehabilitation therapy (CRT), Tai Chi Chuan, aerobic exercise, health education, and psychoeducation. The primary outcome was the effect of meditation on global cognitive performance measured by the Mini-Mental State Examination (MMSE) (21). Secondary outcomes included sleep quality measured by the Pittsburgh Sleep Quality Index (PSQI) (22), health status assessed using the 36-Item Short Form Health Survey (SF-36) (23), and depression evaluated with the Geriatric Depression Scale (GDS). Studies were excluded if they were reviews, reports, abstracts, conference presentations, and empirical studies failing to provide adequate methodological description or accessibility to the full text and complete dataset.

### Selection process

The literature was imported into EndNote version 20 (Clarivate Analytics), and duplicated records were eliminated. Two independent investigators (HT and JWW) screened the titles and abstracts of the retrieved literature, followed by a thorough full-text review based on the predefined eligibility criteria. Any discrepancies between reviewers were resolved through consultation with a third investigator (YHC). Cohen’s Kappa (*κ*) coefficient was calculated to evaluate inter-rater reliability for full-text screening, and the strength of agreement was interpreted using Altman’s criteria (24). The inter-rater agreement between the two reviewers (HT and WWJ) for full-text selection was measured to be excellent (*κ* = 0.862).

### Data extraction

The initial data extraction sheet, piloted by two reviewers (HT and JWW) on non-included articles, was used for data extraction from the selected studies. Both reviewers independently performed the extraction to ensure accuracy and minimize bias. Extracted data included first author, publication year, sample size, study design, participant characteristics, intervention parameters, and outcomes. Discrepancies were resolved by consulting a third reviewer (YHC). All reviewers cross-verified the extracted data sheets before initiating the quality assessment.

### Risk of bias assessment

Two independent authors (JXS and YX) evaluated the risk of bias in individual studies using the Cochrane Risk of Bias 2 (RoB2) tool (25). This tool assesses the randomization process, deviations from intended interventions, missing outcome data, outcome measurement, and selection of reported results. Each study was graded as having a high, low, or unclear risk of bias. Discrepancies between investigators were resolved through consultation with a third reviewer (YHC).

### GRADE certainty assessment

The GRADE system was utilized to rate the certainty of evidence for each outcome. The overall certainty of evidence was categorized into high, moderate, or low levels based on the five key domains of risk of bias, inconsistency, indirectness, imprecision, and other considerations (26).

### Statistical analysis

Data analysis was conducted using R 4.3.1 software. Mean differences (MD) were used for MMSE, PSQI, and QOL scales, while standardized mean difference (SMD) was employed for GDS due to variations in measurement scales across studies. Pre- and post-treatment measurements from all included studies were analyzed. SMD was derived from between-group comparisons of within-group changes from post- to pre-measurements. When the standard deviation (SD) of change scores was unavailable, missing values were calculated using available information, such as the correlation between pre- and post-measurements. If estimations were not feasible, study authors were contacted. Studies were excluded if no response was received within 2 weeks and data remained unobtainable (27, 28). Pooled estimates were evaluated with 95% confidence intervals (CIs), and statistical heterogeneity was assessed using the Chi^2^ test and *I*^2^ statistic. Statistical significance was deemed as *p <* 0.05.


$$\mathrm{MD}={\bar{X}}_{\text{post}}-{\bar{X}}_{\mathrm{pre}}\;\text{or}\;\mathrm{SMD}=\mathrm{MD}\pm \frac{{\bar{X}}_{\text{post}-{\bar{X}}_{\mathrm{pre}}}}{SD_{\text{pooled}}}$$

According to the *Cochrane Handbook for Systematic Reviews*, a fixed-effects model is appropriate only when assuming all effect estimates reflect the same underlying intervention effect (28). Given the substantial differences in intervention designs and control conditions across studies, a random-effects model was utilized when this assumption was unlikely to hold. Multi-arm studies were analyzed separately for each intervention. Subgroup analyses were executed based on intervention duration, meditation type, and different comparators. Publication bias was assessed using funnel plots, complemented by Egger’s (linear regression method) and Begg’s tests (rank correlation method) (29, 30). Sensitivity analyses were performed by excluding studies with high concerns for bias and recalculating pooled estimates to evaluate the robustness of the findings.

## Results

### Characteristics of included studies

**Figure 1:**
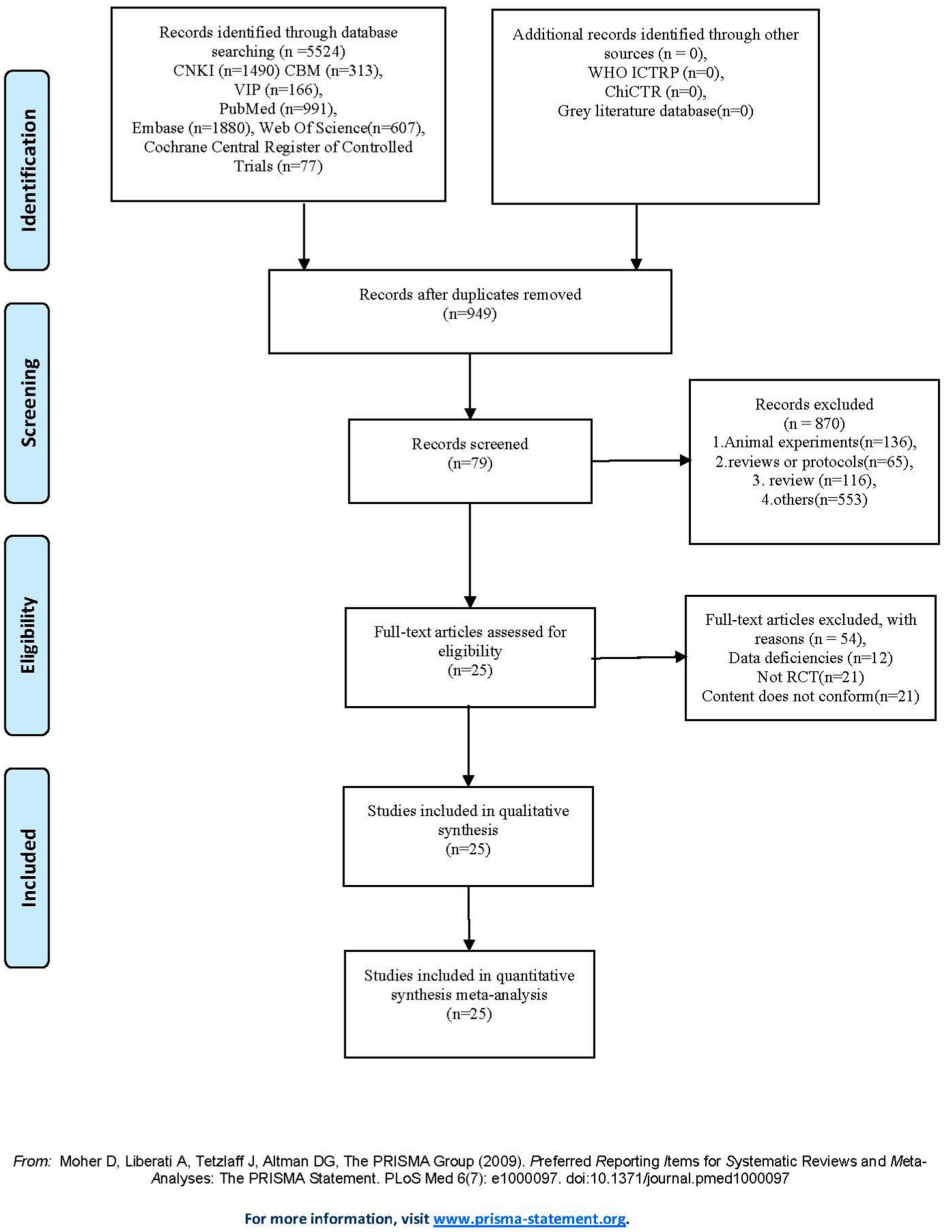
PRISMA flowchart of study screening and selection processes.

A total of 5,523 records were screened, of which 25 RCTs with 2,095 participants were included. Reasons for exclusion at the full-text stage are presented in Figure 1. All included RCTs were parallel-group trials, except for one three-arm study (31). All studies were published between 2013 and 2024 and conducted in multiple countries: one in Canada (32), seven in the United States (5, 14, 33–37), two in the United Kingdom (38, 39), eight in China (40–47), three in Singapore (10, 31, 48), one in Egypt (49), two in Spain (9, 50), and one in the Czech Republic (51). The ages of participants ranged from 53 to 80 years. The meditation interventions were broadly categorized into FAM and DMM. FAM was examined in 19 studies (9, 10, 14, 32–40, 42, 44, 47–51), and DMM was assessed in six studies (5, 35, 41, 43, 45, 46). Specific meditation programs included mindfulness-based cognitive therapy (MBCT) in eight studies (31, 39–42, 45, 47, 49), MBSR in eight studies (9, 38, 43, 44, 46, 48, 50, 51), mindful awareness practice (MAP) in two studies (10, 33), Kabat-Zinn’s in one study (32), Kirtan Kriya in three studies (14, 34, 36), Kundalini yoga in two study (5, 37), and Tibetan Sound Meditation in one study (35). Among the outcomes, global cognitive performance was reported in 14 studies (10, 31, 33, 39–48, 50), sleep quality in six studies (14, 34–36, 46, 49), health status in five studies (5, 14, 34–36), and depression scores in nine studies (9, 10, 31, 32, 37, 38, 46, 49, 51). The duration of interventions varied widely across studies, ranging from 2 to 96 weeks. Most studies reported no significant meditation-related adverse events. Only a few studies mentioned minor adverse events that did not compromise the overall safety and feasibility of the interventions. The characteristics of the studies included in this review are presented in Table 2.

**Table 2:** Characteristics of included studies.

Study, authors (year)	Country	Percentage of patients	Gender (Male/Female)	N (Treatment/Control)	Level of education (years)	Course of disease	Cognitive status	Chronic disease	Treatment group	Control group	Age (mean)	Cognitive status	Disease assessment tool	Description of interventions	Duration of exposure	Adverse events	acquisition time (months)	Outcome of symptoms
Grzenda 2024 (5)	USA	SCD	/	40/39	16.15 ± 1.90/15.72 ± 1.99	/	/	/	Kundalini yoga training	Memory enhancement training	65.45 (9.11)/67.54 (9.30)	MMSE ≤ 23	Self-reported subjective cognitive decline	Kundalini Yoga intervention integrates a structured session of multisensory engagement with daily home practice	For 12 weeks, receive 60-min in-person lessons weekly from a certified KY instructor.	No	0/3/6	BDI, CD-RISC, HAM-A, MFQ, SF-36, CVRF
Larouche, E 2018 (32)	Canada	aMCI	23/22	23/22	13.8 ± 2.8/14.1 ± 3.3	/	/	/	Kabat-Zinn’s MBSR	Psychoeducation-based intervention;	71.4 (7.7)/70.5 (5.6)	/	MoCA; Cognitive Questionnaire	Engage in different mindfulness exercises each week, as detailed in the article	Participants were asked to complete at-home formal meditation practices 6 days a week for 8 weeks in addition to daily informal practices	NA	0/3	GDS-30; GAI-20; WHOQOL-Brief-; FFMQ-22; RRS
Domingo J 2014 (50)	Spain	AD	54/66	30/30/30/30	Unfinished Primary Studies (3 years): 73/Primary Studies (6 years): 17/High school (12 years): 21/University (15 years): 9	1y: 9; 2–3y: 43; 4–5y: 35; >6y: 33	17.00 ± 0.89/16.00 ± 0.93	/	MBSR+UC	CRT/Progressive muscle Relaxation/UC	/	MMSE ≥ 18	DSM-IV criteria	Kirtan Kriya technique: 1. Temporal and spatial orientation; 2. Yoga in the chair; 3. Attention-to-breathing exercise; 4. Body scan; 5. Kirtan Kriya; 6. Guided min dful attention to one of the five senses for 10 min; 7. Psychoeducation on AD	Three weekly group sessions of 90 min for 2 years and a total of 288 sessions in 96 weeks	NA	0/24	MMSE; CAMCOG
Churcher 2017 (33)	USA	Cognitive impairment (Mild [3]/Moderate [28])	16/15	20/11	/	/	15.85 ± 3.68/14.45 ± 4.28	/	MAP+UC	UC	81.30 (9.29)/79.36 (9.91)	MMSE > 18	DSM-IV criteria	Daily home practice (10-Minute Mindful Breathing practice and/or a briefer, 3-Minute Breathing Space)	Twice a week for 5 weeks	No	0/1.25	CSDD; RAID; QOL-AD; MMSE; PSS-13; MBAS
Kim E 2021 (34)	USA	MCI	11/29	20/20	12 years (High school) or less: (4/3); 13–15 years (some college) (6/5); 16+ years (college): (10/12)	/	/	Diabetes (6/3); hypertension (11/7); high cholesterol (7/5); depression (5/2); anxiety (4/3)	Kirtan Kriya	Health education	66.85 (2.14)/61.45 (1.38)		SCD criteria	KK includes a repeated Kirtan or song (singing repetition of the ‘Sa-Ta-NaMa’ mantra) a mudra or physical/motor component (Touching each fingertip to the thumb in sequence with the chant)	12 min daily for 12 weeks (84 practice sessions total)	No	3	MFQ; PSQI; SF-36; QOL
K. Milbury 2013 (35)	USA	Cognitive dysfunction after chemotherapy	/	23/24	/	M: 35.0 ± 14.6/34.33 ± 14.6	/	/	Tibetan Sound Meditation	Wait list control	53.0 (6.6)/54.1 (8.6)	MMSE ≤ 23	FACT-Cogw	TSM program consists of two main components as follows: (i) breathing; (ii) visualization and sound exercises.	Two weeks	No	0/1	FACT-Cog; CES-D; PSQI; BFI; SF-36
Harris A 2016 (37)	USA	MCI	27/52	38/41	/	/	23.9 ± 2.6/24.2 ± 1.7/24.5 ± 1.6	/	Kundalini yoga	Memory enhancement training program	68.1 (8.7)/67.6 (8.0)	MMSE > 24	CDR	(1) tuning in; (2) warm up; (3) breath techniques “Pranayama”; (4) KK; (5) meditation; (6) rest “Shavasana” and closing	Daily 12-min, 60-min per week, and 12 weeks	No	0/3/6	CDR; HVLT; WMS; GDS, AES, CDSC
Piyanee 2019 (48)	Singapore	MCI	14/41	28/27	No degree: (15/20); Primary School: (6/3); Secondary School or ITE: (3/4); Pre-university or Polytechnic: (1/-) University: (2/-) Missing: (1/-)	/	24.61 ± 3.2/24.70 ± 3.89	No chronic health problems (23/22); Visual impairment (3/5); Hearing and visual impairment (1/-); Visual and other impairment (1/-)	MBSR	Health Education	71.26 (5.63)/71.44 (5.97)	/	DSM-IV criteria	The instructors guided the participants to engage in various mindfulness awareness practice techniques: (a) mindfulness of the senses practice; (b) body scan practice; (c) walking meditation practice; (d) movement nature meant practice; (e) Visuo-motor limb tasks, which train the participants in mind–body coordination.	Sessions were conducted weekly for the first 3 months and monthly for the remaining 6 months	NA	0/3/9	GDS-15; GAI-20; MMSE CDR
Natalie L 2021 (38)	UK	SCD	52/95	73/74	13.9 ± 3.8/13.4 ± 3.4	/	28.7 ± 1.2/28.9 ± 1.0	/	MBSR	Health Education	72.1 (7.6)/73.3 (6.2)	/	Criteria for SCD	Building on modifications suggested by Zellner Keller et altogether with a focus on compassion and loving-kindness meditation	Approximately an hour a day, 6 days a week	Twenty-five adverse events were recorded in the trial (CMBAS: 18; HSMP: 7), and 5 of them were considered serious adverse events	0/2/6	State-STAI; GDS-15
Cai 2022 (46)	China	MCI	19/56	38/37	Primary school (4/4); Lower secondary school (4/8); Higher secondary school (19/16); high school (11/9)	/	26.00 (25.00–27.00)/26.00 (25.00–27.50)	Insomnia (9/7)	MBSR	Health Education	80 (8)/80 (10.8)	MMSE: 24 ~ 30	2018 Guidelines for the Diagnosis and Treatment of Dementia and Cognitive Disorders in China	Participants were given a “Walkman” and asked to do 10–45 min of mindfulness practice and homework	1.5 h once a week, for eight courses.	both groups with no deaths or serious adverse events	0/2	Global PSQI; MoCA; VFT; GDS-30; PSS; SAS; MMSE, STT, ISI, AIS, PSS, AVLT, SDMT
Domingo J 2022 (9)	Spain	AD	54/66	30/30/30/30	/	/	/	/	Mindfulness-based Alzheimer’s Stimulation+UC	Usual caretaking-at-home group	/		DSM-5	Mindfulness-based Alzheimer’s Stimulation	Three weekly sessions over years	NA	6/12/18/24	GDS-15; HDRS; NPQ
Ted 2022 (10)	Singapore	MCI	14/41	28/27	5.19 (4.94)/3.44 (4.27)	/	24.59 (3.30)/24.70 (3.89)	/	MAP+UC	Health Education	71.89 (5.94)/70.67 (6.18)	/	DSM-IV criteria	MAP techniques involved: 1) Mindfulness of the senses practice; 2) mindful breathing with body scan practice; 3) movement nature where participants were taught to move with awareness for flexibility, strength and confidence; 4) Visuomotor coordination tasks which trained them in mind–body coordination and lastly; 5) Mindful stretching which aimed to relax their muscles in a mindful manner.	Weekly for 3 months and monthly for the subsequent 6 months	No	0/3/9/60	GDS-15; GAI-20; MMSE-30
Enas 2019 (49)	Egypt	MCI	29/21	24/26	Literate (9/15); intermediate education (10/7); high education (5/4)	/	/	/	MBCT	UC	60–65: 14/17; 65–70: 10/19	/	DSM-IV criteria	MBI consists two parts: 1) sessions concerned with mindfulness meditation practice as sitting meditation, body scan, and walking meditation; 2) sessions emphasized on cognitive stimulation training and memory strategy	Meditation exercise at home for 10 and 30 min, 12 sessions of training program	NA	/	MoCA; PSS; PSQI
Z. Jiayuan2022 (45)	China	Cognitive frailty	37/54	30/31/31	/	/	/	/	MBCT	Tai Chi Chuan/Mindfulness-based Tai Chi Chuan		/	CDR	Four basic forms of meditation practices (body scan, walking meditation, gentle yoga, sitting meditation)	Training for 3 months.	NA	0/6/12	CDR; MMSE-30; TUG; 30-s chair test; SPBB
Kinjal 2021 (31)	Singapore	MCI	34/42	32/27/17	Education (<6 y): 3		26.19 ± 2.73/26.37 ± 3.30	/	MBCT	CRT/UC	67.6 (5.3)/67.1 (3.4)/66.3 (6.7)	MMSE >20	DSM-V	Participants in the intervention delivered in a group setting and engaged in home practice	8 weekly 2 h sessions	NA	0/2	MMSE-30; MoCA-30; GDS-15; MAAS
Kim E 2016 (36)	USA	SCD	9/51	30/30	>12: (3/7); post-high school education (4/11); years of college or more (23/12)	/	/	Diabetes (6/3); hypertension (11/7); high cholesterol (7/5); depression (5/2); anxiety (4/3)	Kirtan Kriya	Music listening program	60.93 (1.56)/60.23 (1.32)	/	SCD criterial	KK program is a multifaceted exercise which engages several areas of the brain. Participants in the intervention delivered in a group setting and engaged in home practice	12 min daily for 12 weeks	No	0/3/6.5	PSS; SF-36; MFQ; PSQI;
Kim E 2019 (14)	USA	SCD	7/46	25/28	Education ≥ 12 y (22/21)	/	/	/	Kirtan Kriya	Music listening program	60.71 (1.38)/60.20 (1.63)	/	MCI criteria	KK program is a multifaceted exercise which engages several areas of the brain. Participants in the intervention delivered in a group setting and engaged in home practice	12 min daily for 12 weeks	No	0/3	PSS; SF-36; MFQ; PSQI
Rafał 2023 (51)	Czech Republic	MCI	7/13	12/8	14.08 ± 3.08/14.63 ± 2.45	/	27.26.82 ± 1.72/27.86 ± 1.36	/	MBSR	CRT	73.83 (7.04)/74.25 (7.25)	/	Diagnosis of MCI	Both formal (body scan, sitting meditation, mindful movement, working with difficulties, meditation with imagination, etc.) and informal practices (bringing mindfulness to routine activities, including short breathing meditation, an analysis of pleasant and unpleasant events and stressful communication, etc.)	8 weekly sessions (2.5 h long) and 1 “retreat in silence” day (6 h long)	NA	0/2/6	Memory Score PVLT, COWAT-FAS; GDS-15; BAI
Mao 2016 (44)	China	AD	140/74	107/107	Missing: (9/12); primary school: (24/19); secondary school: (74/76)	Y: 2.42 ± 1.44/2.66 ± 1.58	18.96 ± 3.24/19.24 ± 3.35	/	MBSR	UC	73.24 (2.12)/72.86 (2.31)	MMSE: 10–26	DSM-IV	Specifically, the routine includes: 1. Body scan; 2. Mindful breathing; 3. Zen sitting; 4. Raisin exercise; 5. Mountain peak meditation; 6. Review of the aforementioned practices.	Three times a day, once in the morning, once in the afternoon, and once in the evening, each session lasting about 30 min. a total of 6 weeks.	NA	0/1.5	MMSE; WMS; MCQ-30; FAQ
Zhao 2022 (43)	China	AD	42/54	48/48	/	/	20.15 ± 3.49/20.36 ± 3.32	/	MBSR+ Chinese medicine	UC	73.16 (4.57)/72.79 (4.61)	MMSE: <27	2018 Guidelines for the Diagnosis and Treatment of Dementia and Cognitive Disorders in China	Specifically, the routine includes: 1. Body scan; 2. Mindful breathing; 3. Mountain peak meditation	1 h each time, 2 times a week; It lasts for 3 months.	NA	0/3	MMSE-30; CAMCOG
Liu 2020 (42)	China	AD	86/100	93/93	Primary School and missing: (48/48); Secondary School: (33/31); University: (12/14)	4.15 ± 1.62/4.06 ± 1.77	/	/	MBCT+UC	UC	70.25 (3.36)/69.95 (3.58)	/	2018 Guidelines for the Diagnosis and Treatment of Dementia and Cognitive Disorders in China	Participants in the intervention delivered in a group setting and engaged in home practice	Once a week for 120 min each time for 8 weeks	NA	0/2	MMSE-30; QOL-AD
Deirdre 2023 (39)	UK	Mild Dementia	5/15	10/10	11.40 ± 2.50/12.1 ± 2.52	/	25.50 ± 3.17/23.50 ± 3.50	/	MBCT+UC	UC	77.80 (10.63)/76.80 (4.96)	/	DSM-IV	Participants in the intervention delivered in a group setting and engaged in home practice	Once a week for 90 min each time for 8 weeks	No	0/2	CSDD; PHQ-9; RAID; GAD-7; QOL-AD; MMSE-30
Zhu 2020 (47)	China	MCI	10/58	34/34	All participants have at least a junior high school education	1.87 ± 0.74/1.76 ± 0.86	13.26 ± 1.66/13.62 ± 1.81	/	MBSR+CRT	CRT	53.82 (4.92)/53.74 (4.95)	MMSE ≤ 24	MMSE	Participants in the intervention delivered in a group setting and engaged in home practice	Once a week for 8 weeks	NA	0/2	MMSE-30; HDS; ADL
Tan 2019 (41)	China	AD	130/134	132/132	/	2.4 ± 2.3/2.2 ± 2.9	19 ± 4/20 ± 4		MBCT	CRT	72.4 (2.2)/72.6 (2.3)	/	AD	Avoid other distractions during 1. meditation; 2. to keep the patient comfortable.3. Zezen 4. Nasal breathing; 5. maintain a relaxed attitude to external interference; 6. Training with less than 68 dB of yoga music and professional instructions.	Three times a day for 8 weeks	NA	0/2	MMSE; MCQ-30
Wang 2019 (40)	China	AD	33/27	30/30	/	/	20.10 ± 2.47/20.32 ± 2.15	/	MBCT+ Aerobic exercise	Aerobic exercise	74.8 (5.4)/75.6 (4.2)	MMSE: 10–24	DSM-IV	Participants in the intervention delivered in a group setting and engaged in home practice	Once a week, 1.5–2 h each time, a total of 8 weeks	NA	0/2/4	MMSE-30; NPI; ADAS-cog

AD, Alzheimer’s Disease; aMCI, Amnestic mild cognitive impairment; CRT, Cognitive rehabilitation therapy; UC, Usual care; SCD, Subjective Cognitive Decline; MBSR, Mindfulness-based stress reduction; MBI, Mindfulness-based interventions; MoCA, Montreal Cognitive Assessment; GDS, Geriatric Depression Scale; GAI, Geriatric Anxiety Inventory; WHOQOL-Brief, World Health Organization Quality of Life Brief scale; RRS, Ruminative Response Scale; FFMQ, Five-Facet Mindfulness Questionnaire; WMS, Wechsler Memory scale; PROMISP, Patient-Reported Outcome Measurement Information System; MMSE, Mini-Mental State Examination, CDS, Clinical Dementia Rating; ADAS-cog, Alzheimer’s Disease Assessment Scale, cognitive subscale; CDR, Clinical Dementia Rating QOL, Quality of life; DSM-IV, The Diagnostic and Statistical Manual of Mental Disorders; CDSS, Cornell Scale for Depression in Dementia; RAID, Rating Anxiety in Dementia Scale; PSS, Perceived Stress Scale; MBAS, Meditation Breath Attention Scores; MFQ, Memory Functioning Questionnaire; PSQI, Pittsburgh Sleep Quality Index; PWBS, Psychological Well-Being Scale; SF-36, Short Form-36; FACT, Function - Cognitive Function Scale; TSM, Tibetan Sound Meditation; HVLT, Hopkins Verbal Learning Test; State-STAI, State–trait anxiety inventory; VFT, Verbal Fluency Test; Auditory Verbal Learning Test; Shape Trail Test; Symbol Digit Modalities Test; OWAT, Oral Word Association Test; APS, Apathy Evaluation Scale; CDSC, Connor–Davidson resilience scale; SPBB, Short Physical Performance Battery.

### Risk of bias assessment and GRADE quality rating

**Figure 2:**
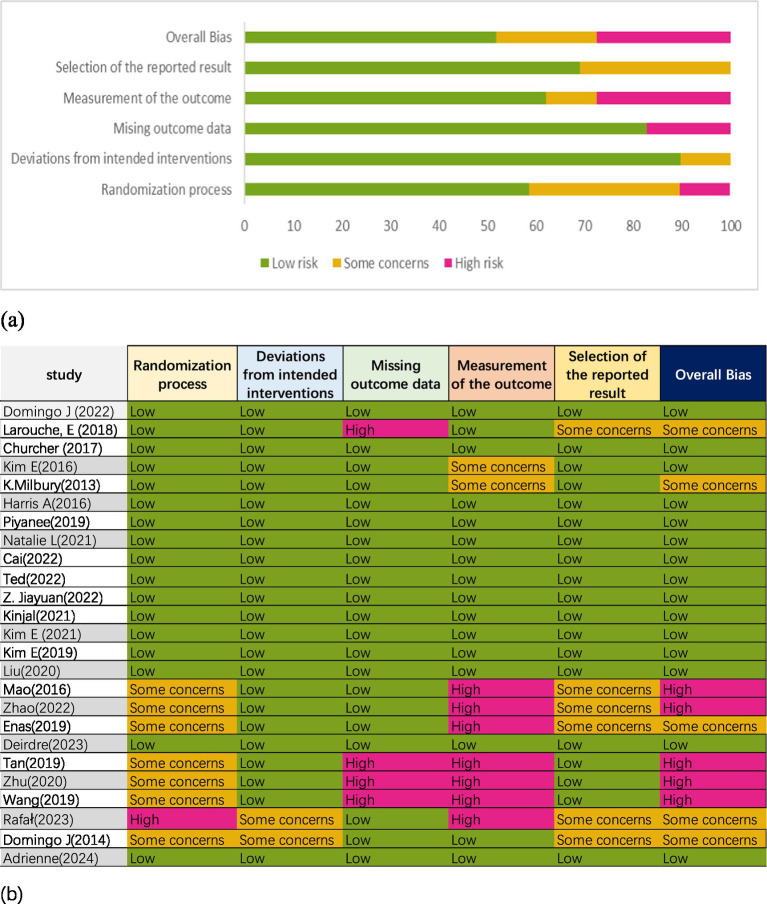
Risk of bias **(a)** graph and **(b)** summary.

**Figure 3:**
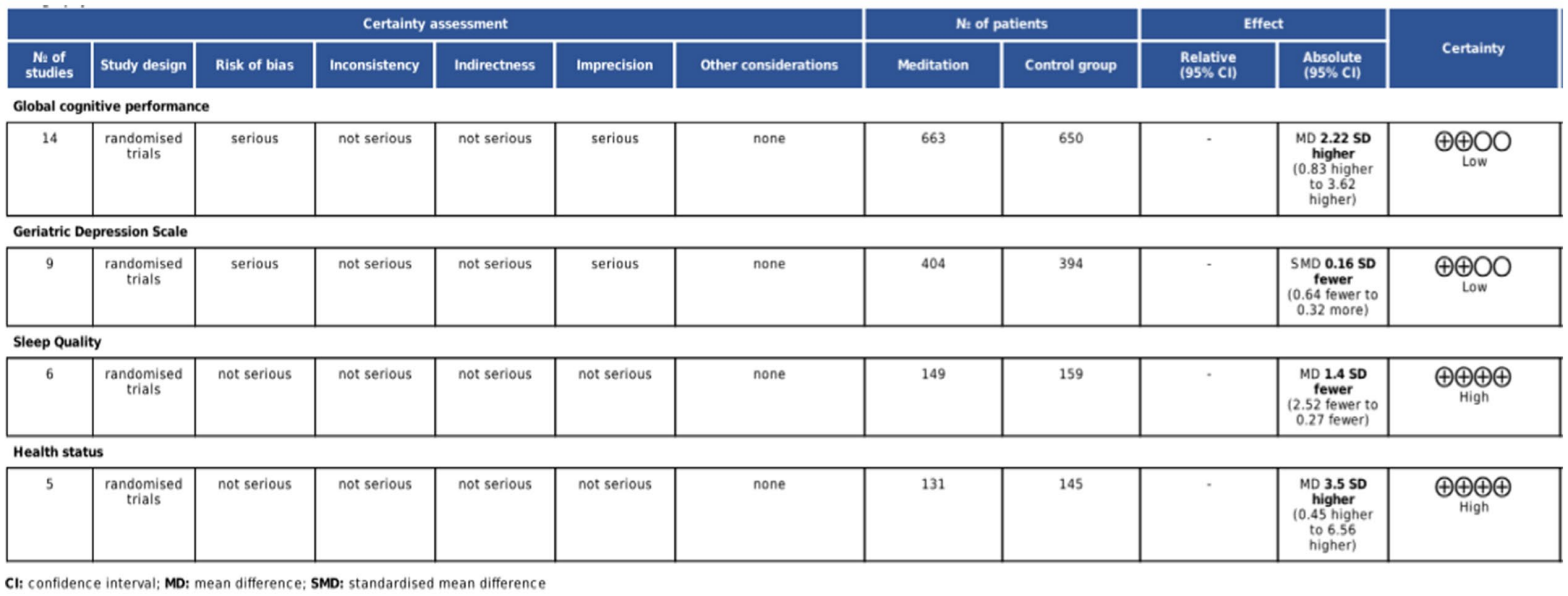
GRADE quality rating.

Seventeen studies described the randomization process (5, 9, 10, 14, 31–39, 42, 45, 46, 48), two studies raised concerns about deviation from intended intervention (50, 51), four studies exhibited a high risk of missing outcome data (32, 40, 41, 47), seven studies presented a high risk in outcome measurement (40, 41, 43, 44, 47, 49, 51), and two had relative risks (31, 36). Six studies had some concerns about the selection of reported results (32, 39, 43, 44, 49, 51). Overall, five studies were classified as high risk (40, 41, 43, 44, 47), five studies had some concerns (32, 35, 49–51), and 15 studies were deemed low risk (5, 9, 10, 14, 31, 33, 34, 36–39, 42, 45, 46, 48). The risk of bias assessment is illustrated in Figure 2. According to the GRADE assessment, the quality of evidence for global cognitive performance and depression was rated as low, whereas the evidence for sleep quality and health status was measured to be of high certainty (Figure 3).

### Primary outcome

#### Global cognitive performance

**Figure 4:**
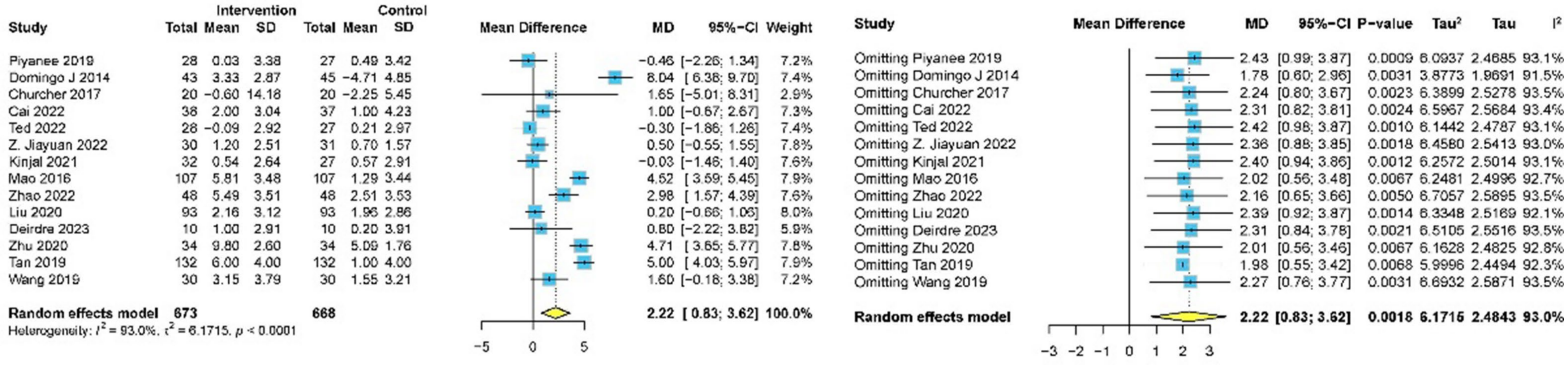
Forest plot for effectiveness of meditation on changes in global cognitive performance.

Fourteen studies involving 1,341 participants assessed global cognitive performance using MMSE (10, 31, 33, 39–48, 50). Meta-analysis of the pooled data demonstrated that meditation exhibited a statistically significant improvement in cognitive performance compared to the control group (MD 2.22, 95% CI: 0.83–3.62, *p* = 0.002) (Figure 4). Subgroup analyses were implemented based on intervention duration, meditation type, and different comparators. Statistically significant improvements in global cognitive performance were observed in 11 studies with intervention lasting less than 6 months (31–33, 39–46) (MD 2.19, 95% CI: 0.94–3.44, *p* < 0.001), while no significant effect was found in three studies with intervention lasting more than 6 months (10, 48, 50) (MD 2.43, 95% CI: −3.07 to 7.93, *p* = 0.387). Subgroup analysis by meditation type revealed statistically significant improvements in both FAM (10, 33, 39, 40, 42, 44, 47, 48, 50) (MD 2.39, 95% CI: 0.39–4.40, *p* = 0.019) and DMM (31, 41, 43, 45, 46) (MD 1.92, 95% CI: 0.06–3.79, *p* = 0.043). For different comparators, meditation was superior to usual care (31, 33, 39, 42–44, 50) (MD 2.70, 95% CI: 0.38–5.03, *p* = 0.023) and CRT (41, 47) (MD 4.87, 95% CI: 4.16–5.58, *p* < 0.001). No significant difference was observed when meditation was compared with aerobic exercise (40) (MD 1.60, 95% CI: −0.18 to 3.38, *p* = 0.080), health education (10, 46, 48) (MD 0.85, 95% CI: −0.87 to 1.05, *p* = 0.862), and Tai Chi Chuan (45) (MD 0.50, 95% CI: −0.55 to 1.55, *p* = 0.350) (Table 3).

**Table 3:** Subgroup analysis of meditation effectiveness on global cognitive performance.

Subgroup type		Number	Sample size	MD (95% CI)	*I*^2^ (%)
Intervention duration	>6 months	3	99/99	2.43 (−3.07, 7.93)	74.7
	<6 months	11	574/569	2.19 (0.94, 3.44)	91.7
Meditation type	DMM	5	280/275	1.92 (0.06, 3.79)	92.7
	FAM	9	393/393	2.39 (0.39, 4.40)	93.9
Different comparators	UC	7	353/350	2.70 (0.38, 5.03)	89.3
	Tai Chi Chuan	1	30/31	0.50 (−0.55, 1.55)	/
	CRT	2	166/166	4.87 (4.16, 5.88)	0
	Aerobic exercise	1	30/30	1.60 (−0.18, 3.38)	/
	Health Education	3	94/91	0.09 (−0.88, 1.05)	0

CRT, Cognitive rehabilitation therapy; DMM, dynamic mindfulness meditation; FAM, focused attention meditation; MD, Mean differences; UC, Usual care.

**Figure 5:**
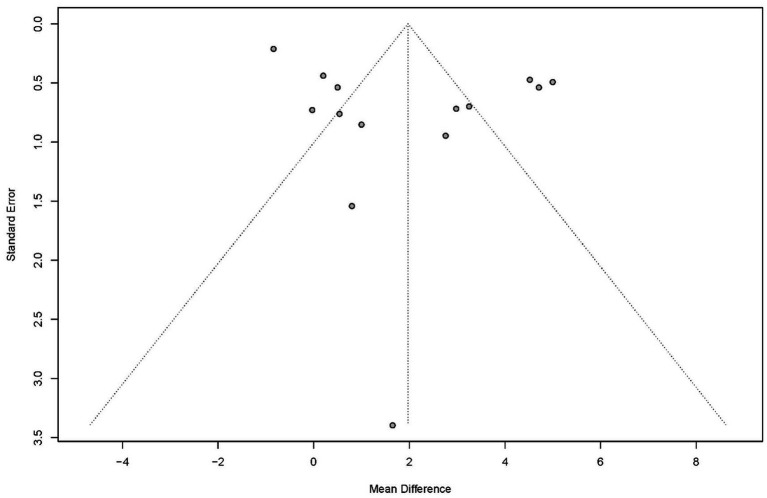
Funnel plot for publication bias in global cognitive performance outcome.

Publication bias for global cognitive performance was evaluated using funnel plots (Figure 5), and no evidence of bias was indicated by Egger’s test (*p* = 0·619) and Begg’s test (*p* = 0·869). Sensitivity analysis for global cognitive performance confirmed high heterogeneity (*I*^2^ = 95%). Sequential exclusion of individual studies revealed minimal impact on the overall effect size and heterogeneity. The 14 study-specific MD ranged from 2.22 (95% CI: 0.83–3.62) to 1.78 (95% CI: 0.60–2.96), with heterogeneity slightly decreasing to *I*^2^ = 91.5%. Overall, the effect size remained statistically significant (*p* < 0.05), indicating the robustness of the findings.

### Secondary outcomes

#### Sleep quality

**Figure 6:**
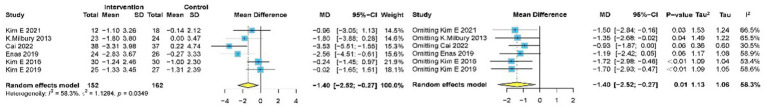
Forest plot for effectiveness of meditation on changes in sleep quality.

Six studies (14, 34–36, 46, 49) with 316 participants assessed sleep quality using PSQI. Meta-analysis of the pooled data demonstrated that meditation significantly improved the quality of sleep, as evident by the decreased PSQI (MD −1.40, 95% CI: −2.52 to −0.27, *p* = 0.015) (Figure 6).

Sensitivity analysis confirmed high heterogeneity (*I*^2^ = 58.3%), and sequential exclusion of individual studies showed minimal impact on the overall effect size and heterogeneity. The six study-specific MD ranged from −1.40 (95% CI: −2.52 to −0.27) to −0.93 (95% CI: −1.87 to 0.00), with heterogeneity slightly decreasing to *I*^2^ = 30.5%.

#### Health status

**Figure 7:**
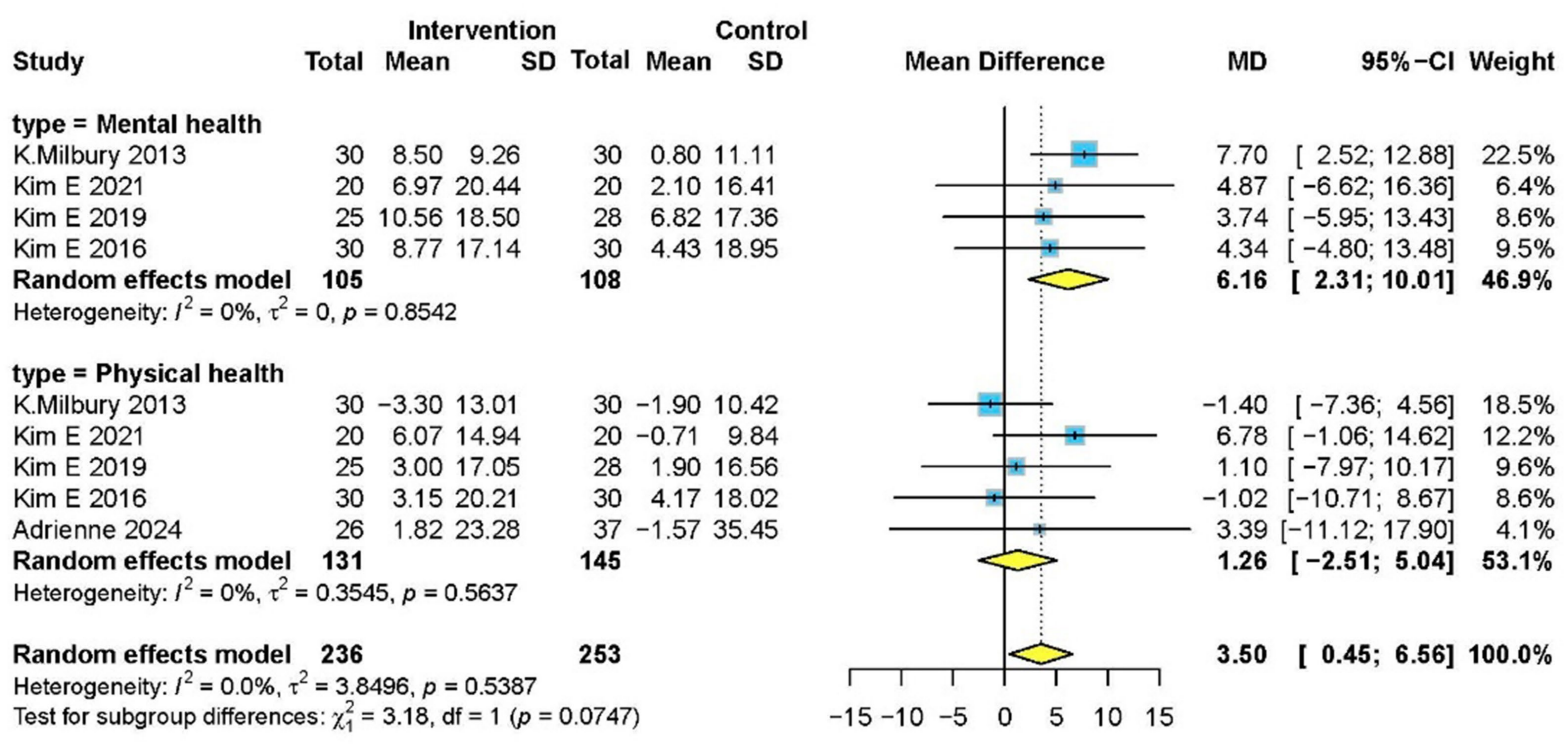
Forest plot for effectiveness of meditation on changes in health status.

Five studies (5, 14, 34–36) with 276 participants evaluated health status using SF-36. Meditation significantly improved overall health status (MD 3.50, 95% CI: 0.45–6.56, *p* = 0.020) and mental health subdomain (MD 6.16, 95% CI: 2.31–10.01, *p* = 0.010), though no significant effect was observed in physical health subdomain (Figure 7).

#### Depression

**Figure 8:**
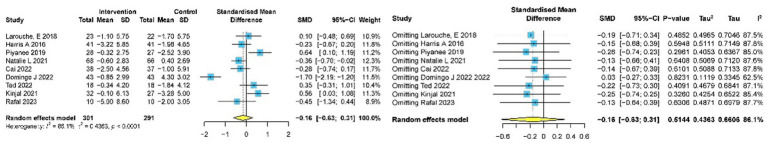
Forest plot for effectiveness of meditation on changes in depression.

Nine studies (9, 10, 31, 32, 37, 38, 46, 48, 51) involving 592 participants assessed the impact of meditation on depression using GDS. Pooled data indicated no significant reduction in depression (SMD −0.16, 95% CI: −0.63 to 0.31, *p* = 0.514) (Figure 8). Sensitivity analysis reduced heterogeneity from *I*^2^ = 86.1% to *I*^2^ = 62.5%, with an adjusted effect size of SMD = 0.03 (95% CI: −0.27 to 0.33). No publication bias was detected by Egger’s test (*p* = 0.374) and Begg’s test (*p* = 1.000).

## Discussion

Meditation has emerged as a scalable and cost-effective non-pharmacological therapeutic intervention, extending beyond clinical settings into community-based and older adult care frameworks (15, 44). This meta-analysis systematically evaluated the effectiveness of meditation across multiple domains, such as global cognitive performance, sleep quality, health status, and depression, in older adults with SCD, MCI, and AD. The findings demonstrated that meditation significantly improved global cognitive performance, sleep quality, and health status outcomes in this population. These results are consistent with prior cohort studies (52, 53), underscoring the potential of integrative public health approaches in mitigating cognitive decline. By synthesizing contemporary evidence and incorporating multidimensional outcome assessments, this study further highlighted the utility of meditation as a pragmatic and adjunctive therapeutic strategy for addressing cognitive decline in aging populations.

MMSE, a commonly used cognitive assessment scale, was employed in this study to evaluate the effectiveness of meditation on global cognitive performance. Pooled analysis showed that meditation significantly improved cognitive performance among individuals with SCD, MCI, and AD, consistent with previous studies demonstrating substantial cognitive enhancement from meditative practices (54). Subgroup analysis based on intervention duration suggested that meditation lasting less than 6 months yielded positive outcomes, which may provide insights for optimizing treatment duration in future practice. When stratified by meditation type, both FAM and DMM demonstrated notable memory-related benefits. FAM has been reported to foster mental focus and stability, improve attention, and reduce distractions, while DMM incorporates physical movement and may enhance physical function and alleviate stress through increased body awareness. Patients may benefit from selecting meditation modalities in accordance with their individual needs to maximize the desired outcomes (6). Additionally, subgroup analysis of comparators indicated that meditation outperformed both usual care and CRT in improving global cognitive performance. However, given the heterogeneity and limited sample sizes across included studies, these findings might be interpreted with cautious.

This study also provided robust evidence that meditation significantly enhanced sleep quality measured by PSQI and mental health subdomains of SF-36 in patients with SCD, MCI, and AD. These benefits may be attributed to the capacity of meditation to reduce ruminative thinking and enhance emotional regulation (55). Although no statistically significant improvements were observed in the physical health subdomain of SF-36, the mental health gains aligned with the growing recognition of meditation as a complementary therapy for insomnia and psychosocial wellbeing (8, 56). In the present study, depression scores showed no statistically significant differences between meditation and control groups. This may be associated with subclinical baseline depression levels in the included RCTs, potentially limiting the measurable impact of interventions. Future studies targeting cognitive decline populations with moderate-to-severe depression may help further elucidate the potential benefits of meditation in this domain (38, 46, 48).

While this study highlights the potential benefits of meditation for older adults with SCD, MCI, and AD, further explorations are still warranted from the perspective of the RE-AIM framework. The promotion of interventions in public health settings depends on their ‘Reach’ and ‘Implementation’ quality. The implementation quality of meditation may vary significantly across diverse populations, particularly among older adults, where such challenges as limited accessibility, variable adherence, and the need for contextual adaptability may arise (16). Additionally, the absence of long-term follow-up data in most studies underscores the need for future research to incorporate regular assessments and supportive measures to evaluate sustained effectiveness over extended periods further (57). Addressing these issues may facilitate the applicability of meditation in clinical and community settings and ensure its sustained effectiveness.

## Strengths and limitations

The strengths of this study are as follows: (1) it covers the neurodegenerative continuum of SCD, MCI, and AD, while incorporating the most recent evidence to provide a contemporary synthesis of the therapeutic potential of meditation; (2) the systematic search strategy was designed to include both English and Chinese language databases, mitigating linguistic bias and enhancing the global applicability of findings; (3) the study employed multidimensional outcome assessments, yielding a more comprehensive evaluation of meditation impact that transcends the narrow cognitive focus of earlier reviews; and (4) the application of the GRADE ensured transparent and standardized appraisal of evidence certainty and might reinforce the robustness and reliability of outcomes.

However, several limitations warrant consideration: (1) although the search strategy prioritized database comprehensiveness, the exclusion of gray literature and manual reference searches might have introduced selection bias; (2) small sample sizes in some included studies might limit statistical power, though sensitivity analyses confirmed that small-sample effects did not drive heterogeneity; (3) while MMSE is widely used and highly accepted among health professionals, it has limited responsiveness over time and may carry a high risk of bias in detecting subtle cognitive changes (58, 59); (4) substantial heterogeneity remained persistently evident in MMSE-measured global cognitive performance, which might limit the reliability of pooled estimates and undermine the interpretability of the findings; (5) heterogeneous outcome assessment tools employed across the included RCTs necessitated focusing on certain selected subset, which might result in incomplete findings by excluding relevant data captured by alternative instruments; and (6) technology-enhanced meditation interventions have shown emerging promise for populations at risk of cognitive decline (60, 61), their absence in this study may limit the generalizability of the findings.

## Implications for further research

For future research, further investigation into the effects of meditation on patients with cognitive decline remains warranted. Conducting multi-center, large-sample, high-quality clinical trials is crucial to further validate the positive effects of meditation and assess its applicability in patients with cognitive decline. Special attention should be paid to how meditation may impact patients at various stages of cognitive decline, as the effects may differ depending on disease progression. To better understand the long-term benefits of meditation, future studies should incorporate extended follow-up periods to evaluate its sustained effects. Moreover, given the complex nature of cognitive health, interdisciplinary collaboration will be pivotal. Future research should foster cooperation across fields, such as psychology, neurology, and clinical medicine, to decipher the biological mechanisms underlying meditation effects and enhance its clinical applications in public health.

## Conclusion

This study highlights the potential of meditation as an effective adjunct approach for older adults with SCD, MCI, and AD, yielding significant improvements in global cognitive performance, sleep quality, and health status. However, given the limitation in evidence quality, heterogeneity, and sample sizes across studies, these findings should be interpreted with caution. More large-scale and well-designed high-quality RCTs with long-term follow-ups are warranted to validate the effectiveness of meditation further and decipher its underlying mechanisms.

## Data Availability

All datasets generated for this study are included in the article/supplementary material.

## Footnotes

^1^ https://www.alz.org/

^2^ http://dx.doi.org/10.1097/MD.0000000000019313
